# Breast ultrasound in the management of gynecomastia in Peutz–Jeghers syndrome in monozygotic twins: two case reports

**DOI:** 10.1186/1752-1947-8-440

**Published:** 2014-12-18

**Authors:** Graziella Di Grezia, Tiziana Romano, Francesco De Francesco, Francesco Somma, Gaetano Rea, Roberto Grassi, Gianluca Gatta

**Affiliations:** 39 Department of Clinical and Experimental Medicine “F. Magrassi, A. Lanzara”, Section of Radiology and Radiotherapy, Second University of Naples, Naples, Italy; 40 Pediatric Department, Second University of Naples, Naples, Italy; 41 Department of Orthopedic, Traumatologic, Rehabilitative, and Plastic-Reconstructive Sciences, Second University of Naples, Naples, Italy; 42 Radiology Department, Monaldi Hospital, Ospedale dei Colli, Naples, Italy

**Keywords:** Breast ultrasound, Gynecomastia, Peutz–Jeghers, Subcutaneous mastectomy

## Abstract

**Introduction:**

Peutz–Jeghers syndrome is an autosomal dominant disease with incomplete penetrance and variable expression caused by germline mutation of serine threonine kinase 11/liver kinase B1; it is characterized by hamartomatous polyps in the gastrointestinal tract, mucocutaneous melanin pigmentation, and increased predisposition to neoplasms. In Peutz–Jeghers syndrome, bilateral Sertoli cell testicular tumors cause endocrine manifestations including gynecomastia and feminization.

This study aimed to assess the role of breast ultrasound in the evaluation of the effectiveness of an innovative surgical approach.

**Case presentation:**

This report presents a pair of European 9-year-old identical male twins with Peutz–Jeghers syndrome, bilateral prepubertal gynecomastia, and testicular multifocal calcifications. Both twins were treated with anastrozole for 2 years. After finishing treatment, both underwent subcutaneous mastectomy performed by the “modified” Webster technique. Breast examination and ultrasound were performed before and after the pharmacological and surgical treatment. A breast ultrasound scan before surgery showed bilateral gynecomastia in both patients. No solid nodular or cystic formations were present on either side. After pharmacological therapy and surgical glandular removal, a breast examination showed a significant reduction in breast volume; 1 year after surgery, a breast ultrasound scan of both patients showed a total absence of glandular parenchyma, with muscle planes well represented.

**Conclusions:**

Breast examination and ultrasound have proved to be a valid approach in the assessment of the treatment of prepubertal gynecomastia because they allow the efficacy of the pharmacological and surgical treatment to be evaluated in a multidisciplinary approach to one of the most frequent endocrine manifestations of Peutz–Jeghers syndrome.

## Introduction

Peutz–Jeghers syndrome (PJS) is an autosomal dominant disease with incomplete penetrance and variable expression [1, 2] caused by germline mutation of serine threonine kinase 11 (*STK11*/liver kinase B1, *LKB1*) [3, 4].

It is characterized by increased predisposition to neoplasms [5] and hamartomatous polyps in the gastrointestinal tract, and mucocutaneous melanin pigmentation with high phenotypic variability. In PJS, high aromatase activity [6] causes oversecretion of estrogen by bilateral Sertoli cell testicular tumors (large-cell calcifying tumors) with advanced bone age, feminization and gynecomastia.

Gynecomastia is a benign condition characterized by enlargement of the male breast due to proliferation of glandular tissue; it is common in normal males during the neonatal period, at early puberty, and with increasing age. Prepubertal gynecomastia is characterized by the presence of palpable uni- or bilateral breast tissue in boys without other signs of sexual maturation. It could be related with excessive estrogen production by adrenal or testicular tumors [7–9], also in rare syndromes such as PJS, congenital adrenal hyperplasia*,* or overexpression of aromatase or to the use of drugs that affect androgen and estrogen production and metabolism. This case report describes the role of breast ultrasound in the surgical management of prepubertal gynecomastia and subsequent follow-up in monozygotic twins with PJS and bilateral multifocal testicular calcifications.

## Case presentation

A pair of European 9-year-old identical male twins (patients 1 and 2) with PJS presented with bilateral progressive prepubertal gynecomastia over the course of 1 year. The family history showed that their father had PJS but no history of gynecomastia or testicular calcification. Neither mutations nor deletions where found in the tumor suppressor gene *LKB1*/*STK11*, which is responsible for approximately 60% of PJS cases.

The twins arrived at our Department in 2008. A physical examination showed two boys with pigmented lesions of the lips and bilateral gynecomastia with a diameter of 9cm in patient 1 and 7cm in patient 2, corresponding to a female Tanner stage B3. Their testicular volume was 4mL bilaterally. The boys’ penises were infantile, and they had no pubic or axillary hair (pubic hair, PH1; genitalia development, G1).

The height of patient 1 was 129.2cm (25th percentile), with a growth velocity of 7cm/year (90th percentile for age) and normal weight for height. The height of patient 2 was 125.5cm (10 to 25th percentile), with a growth velocity of 6cm/year (75 to 90th percentile) and normal weight for height. The target height was 173cm (−0.7 Standard Deviation Score, SDS).

### Hormonal treatment

Hematic levels of sexual hormones were constantly verified with specific reference to luteinizing hormone (LH), follicle-stimulating hormone (FSH), prolactin, testosterone, estrone and estradiol.

Baseline endocrine investigations in patients 1 and 2 showed normal prepubertal serum concentrations of testosterone, FSH, LH and dehydroepiandrosterone sulfate, as well as slightly elevated levels of estradiol, with normal levels of estrone.

Both boys were treated with the third-generation aromatase inhibitor, anastrozole, starting dose of 1mg orally once daily. The decision to treat the boys with the aromatase inhibitor anastrozole had been implemented to reduce gynecomastia and to prevent the accelerating effect of estrogen excess on skeletal maturation.

Samples were obtained before the beginning of the anastrozole treatment, then after 1 and 2 years of treatment, during 2-years follow-up evaluation, before and after 3 months subcutaneous mastectomy surgery.

During the period of anastrozole treatment, a reduction of gynecomastia was observed more in one twin than in the other. In particular, during the first year of treatment, growth velocity decreased from 7 to 3cm and gynecomastia decreased from 9 to 4cm in diameter in patient 1, whereas growth velocity decreased from 6.6 to 3cm/year and gynecomastia decreased from 7 to 3cm in diameter in patient 2.

During the second year of treatment, no changes in gynecomastia occurred, and growth velocity reverted to normal values for age in patient 1 (5cm/year), whereas it remained below the normal value for age in patient 2 (3.5cm/year).

After 2 years, a sharp reduction in growth velocity occurred for both patients (although it was more pronounced in patient 2, with a growth velocity of 1.6cm/year, 3rd percentile), which prompted a reduction in the anastrozole dose and then its withdrawal at the age of 11.5 years.

**Figure 1:**
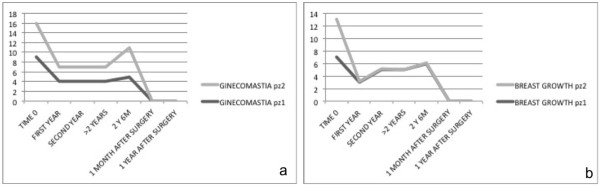
**a) breast volume, b) breast growth in patients 1 and 2 at enrollment, first year, second year, after 2 years of medical treatment, at 2 years and 6 months (after medical treatment interruption, before surgical treatment), and 1 month and 1 year after surgery.** In the vertical line we consider years of patients, from 0 to 18 years old. pz, patient; M, months; Y, years.

After a washout period of 6 months, clinical reevaluation of both twins showed regrowth of gynecomastia, corresponding to a female Tanner stage B3, with resumed tenderness of the breast to palpation (Table 1, Figure 1).

**Table 1:** **Breast ultrasound monitoring of gynecomastia and growth before treatment (enrollment), during 2 years of pharmacological treatment (first and second year), at pharmacological interruption (>2 years), and before and after surgical removal**

		Enrollment	Pharmacological treatment		Pharmacological interruption	Before surgery	1 month after surgery	1 year after surgery
			First year	Second year	>2 years	2 years 6 months		
*Breast growth (cm/year)*	Patient 1	7	3	5	5	6	0	0
	Patient 2	6.6	3	3.5	1.6	3.6	0	0
*Gynecomastia (cm)*	Patient 1	9	4	4	4	5	0	0
	Patient 2	7	3	3	3	6	0	0

### Bone age

To determine bone age, X-rays of each patient’s nondominant hand and wrist were analyzed by the TW2 method before anastrozole treatment was started, and after 1 year of treatment and 2 years of treatment.

Bone age was 11.2 years in patient 1 and 10.9 years in patient 2, with an advance of approximately 2.5 years and an adult height prognosis of 157cm (−3 SDS) for patient 1 and 158.7cm (−2.8 SDS) for patient 2 (both below the target height).

### Ultrasound findings

Ultrasound was performed using a Logiq S6 scanner (GE Healthcare, Waukesha, WI, USA) with a multifrequency matrix-array linear transducer (7 to 14MHz) and a multifrequency convex transducer (3.5 to 5Mhz).

A breast ultrasound to assess palpable masses was performed during and after pharmacological treatment, before and after 1 month and 1 year from subcutaneous mastectomy surgery. The examinations showed bilateral gynecomastia with typical hyperechoic fibroglandular tissue. No solid nodular or cystic formations were present on either side.

An abdominal ultrasound scan of their adrenal glands and a testicular ultrasound scan were performed to analyze the presence of estrogen-producing adrenal tumor; a testicular ultrasound scan showed bilateral multifocal calcifications.

### Surgical treatment

At the age of 13 years, the twins underwent gynecomastia surgical treatment, using the Webster technique. A skin incision was made below the areola edge and the adipose and glandular tissues were significantly removed under direct vision from the skin at the top and from the pectoral muscle at the bottom; the removed breast tissue was sent to a pathologist.

The result was radical surgery (thus avoiding recurrence) and a good aesthetic result.

**Figure 2:**
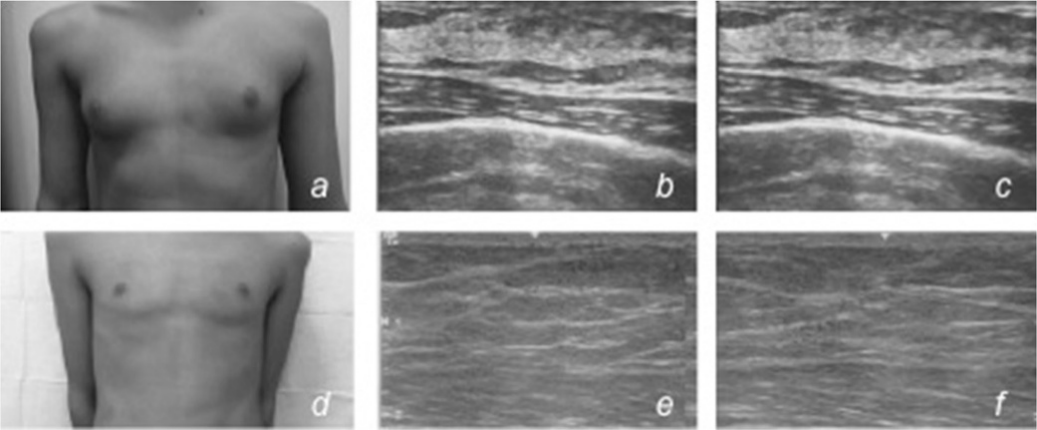
**Patient 1.** Clinical preoperative breast view **(a)** and breast ultrasound **(b,c)** scan showing bilateral gynecomastia. No solid nodular or cystic formations are present on either side; clinical postoperative breast view **(d)** and breast ultrasound **(e,f)** scan 1 year after surgery showing a total absence of glandular parenchyma, with muscle planes well represented.

**Figure 3:**
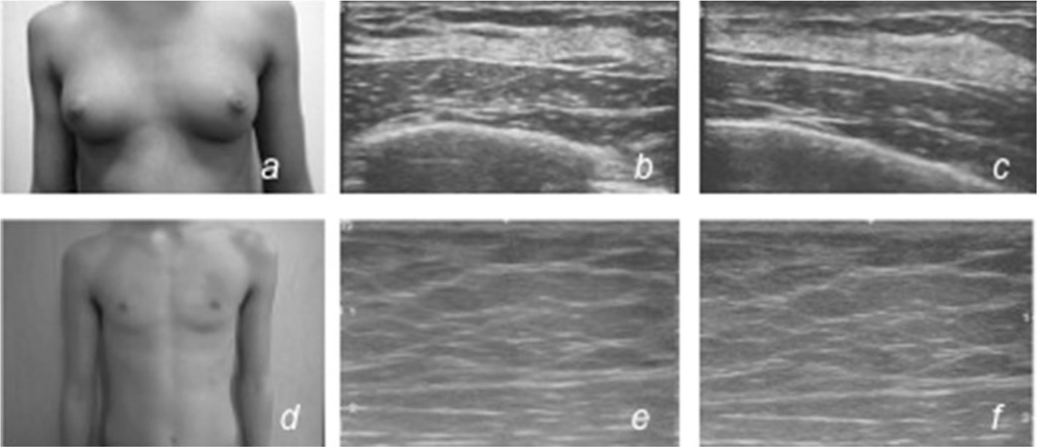
**Patient 2.** Clinical preoperative breast view **(a)** and breast ultrasound **(b,c)** scan showing bilateral gynecomastia. No solid nodular or cystic formations are present on either side; clinical postoperative breast view **(d)** and breast ultrasound **(e,f)** scan 1 year after surgery showing a total absence of glandular parenchyma, with muscle planes well represented.

In both boys, an advancement flap of adipose tissue below the nipple–areola complex was placed, in consideration of the underlying disorder, to avoid leaving a residual subareolar glandular parenchyma (Figures 2 and 3).

Although the glandular tissue had been completely removed, this technique allowed a good aesthetic result, thus avoiding depression of the nipple–areola complex, which would remain responsive to the circulating estrogen.

Both patients were satisfied with the result and had no functional impairment of their arms or reduction in nipple–areola complex sensitivity. Wound healing was completed in 10 days, and scars were almost undetectable in both cases. The excessive removal of parenchyma did not result in skin irregularities, avoiding any unnecessary subsequent treatment. Both patients received postoperative pain management with painkillers according to their weight. The clinical course had no irregularities. No surgical infections or hematomas were reported. Although the breast gland had been totally removed, both patients had good cutaneous retraction and no nipple or areola necrosis or deformity, as confirmed by ultrasound picture control.

Histologic findings confirmed their condition to be benign. After surgical glandular removal, a breast examination showed a significant reduction in breast volume; 1 year after surgery, a breast ultrasound scan of both patients showed a total absence of glandular parenchyma, with muscle planes well represented.

A compressive medication was applied after surgery with a chest bandage for 4 weeks.

Analgesics were prescribed only in case of pain. Both boys were observed by surgeons 1 and 4 weeks after surgery for removal of the bandage and to exclude the onset of hematomas, seromas, skin infections, keloids and pain.

### Follow-up

After surgery, the twins were followed for an additional year for evaluation of sex hormone levels, growth curve and breast size.

## Discussion

Gynecomastia is a multifactorial disease, primarily dependent on the balance between free estradiol and free testosterone. Estrogen biosynthesis results from the production of androgens from steroid precursors; therefore, an excess of endogenous estrogen can be a result of an increase in substrate, aromatase activity, or both.

An increase in the level of estrogen is the most common cause of imbalance. The most common causes of gynecomastia are endocrinopathies, neoplasm-producing estrogens, human chorionic gonadotropin or aromatase, hyperaromatase syndrome [6] and only few cases of idiopathic prepubertal disease. The frequencies of some remaining causes have been estimated as follows: cirrhosis (8%), primary hypogonadism (8%), testicular tumors (3%), secondary hypogonadism (2%), hyperthyroidism (1.5%), and renal disease (1%).

Testicular cancer (associated with both germ cell tumors and non-germ cell tumors) accounts for 1 to 1.5% of all male neoplasms. Although PJS represents one of the genetic conditions predisposing to these types of tumors, it remains a rare condition [10–13]. Our two patients with PJS with large-cell calcifying Sertoli cell tumors had prepubertal gynecomastia for approximately 1 year (Tanner stage 3) and had advanced bone age from the increased level of serum estradiol.

The treatment of prepubertal gynecomastia depends on its etiology. Medical therapy with an aromatase inhibitor alone is not effective in reversing prepubertal gynecomastia. The initial florid stage, characterized by ductal proliferation, can respond to medical treatment but, subsequently, glandular hyperplasia is replaced by progressive fibrosis and hyalinization; when it causes painful or psychological disturbance, surgical removal of the breast glandular tissue is indicated [10, 14].

However, anastrozole represents the first noninvasive step of treatment in PJS and may be useful for delaying surgery in very young patients to achieve an optimal aesthetic result. Plastic surgeons used a standard mastectomy through a semicircular incision within the margin of the pigmented area of the areola to conceal the scar. The standard technique included positioning a 5- to 7-mm pad under the nipple to prevent dermal adherence to the pectoralis fascia. To prevent possible recurrence, they totally removed the gland and set up an advancement flap of adipose tissue to prevent adhesion of the nipple to the pectoralis fascia and its unaesthetic depression.

In conclusion, gynecomastia is a multifactorial disorder, and a correct evaluation must be performed to distinguish physiologic and pathologic causes. The history must include testicular assessment symptoms of hepatic disease and alcohol consumption as well as family history. Physical examination and a routine biochemical profile permit an acknowledgement and recognition of most underlying causes.

## Conclusions

Although severe gynecomastia is a rare condition in the pediatric age, competence and expertise is necessary for the treatment of these patients. Breast examination and ultrasound have proved to be a valid approach in the assessment of the treatment of prepubertal gynecomastia because they allow the efficacy of the pharmacological and surgical treatment to be evaluated in a multidisciplinary approach to one of the most frequent endocrine manifestations of PJS.

## Consent

Written informed consent was obtained from the patients’ mother for publication of this case report and accompanying images. A copy of the written consent is available for review by the Editor-in-Chief of this journal.

## Abbreviations

FSH: Follicle-stimulating hormone
LH: Luteinizing hormone
PJS: Peutz–Jeghers syndrome
SDS: Standard Deviation Score.
